# Embedded Spherical Localization for Micro Underwater Vehicles Based on Attenuation of Electro-Magnetic Carrier Signals

**DOI:** 10.3390/s17050959

**Published:** 2017-04-26

**Authors:** Daniel-André Duecker, A. René Geist, Michael Hengeler, Edwin Kreuzer, Marc-André Pick, Viktor Rausch, Eugen Solowjow

**Affiliations:** Institute of Mechanics and Ocean Engineering, Hamburg University of Technology, Hamburg 21073, Germany; daniel.duecker@tuhh.de (D.-A.D.); andreas.geist@tuhh.de (A.R.G.); michael.hengeler@tuhh.de (M.H.); kreuzer@tuhh.de (E.K.); pick@tuhh.de (M.-A.P.); viktor.rausch@tuhh.de (V.R.)

**Keywords:** localization, micro autonomous underwater vehicles, swarm robotics, software- defined radio

## Abstract

Self-localization is one of the most challenging problems for deploying micro autonomous underwater vehicles (μAUV) in confined underwater environments. This paper extends a recently-developed self-localization method that is based on the attenuation of electro-magnetic waves, to the μAUV domain. We demonstrate a compact, low-cost architecture that is able to perform all signal processing steps present in the original method. The system is passive with one-way signal transmission and scales to possibly large μAUV fleets. It is based on the spherical localization concept. We present results from static and dynamic position estimation experiments and discuss the tradeoffs of the system.

## 1. Introduction

Micro autonomous underwater vehicles (μAUVs) are currently a prominent research topic and are expected to gain more importance in the future, especially for applications in confined liquid-filled tanks, e.g., in process engineering. However, current self-localization approaches for μAUVs pose great challenges and do not provide satisfactory and reliable performance yet, which is a key capability for autonomous operations.

**Figure 1:**
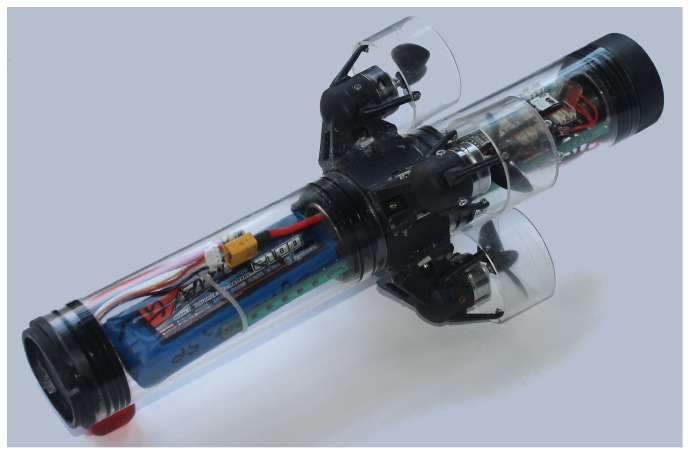
Photo of HippoCampus. A μAUV which is 35 cm long and suitable for multi-robot operations in liquid-filled tanks [2].

**Figure 2:**
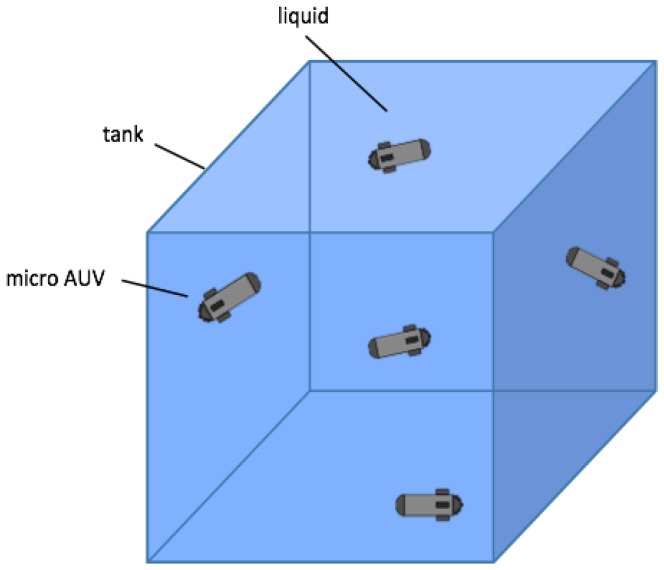
Illustration of an application scenario for a fleet of μAUVs. A confined tank contains a liquid with an underlying concentration field of interest. The μAUVs perform adaptive measurements to collect information and to infer the state of the concentration field.

Advances in the area of microelectronics are continuously leading to the miniaturization and cost decrease of hardware, such as motor controllers and sensor suits. This enables groundbreaking possibilities for the development of novel μAUVs. Underwater vehicles are usually termed μAUVs if their characteristic length is less than 50 cm. Recent examples are the Avexis submersible [1] and the HippoCampus robot [2]; the latter is shown in Figure 1. Micro AUVs are becoming increasingly interesting for industrial applications where they are operated in confined liquid-filled tanks. Examples include nuclear storage ponds [1], tanks in process engineering or wastewater treatment. Figure 2 illustrates a potentially large fleet of μAUVs operating in a liquid-filled tank. Tank environments considered in this work have length scales of approximately 3 m to 25 m. While the mentioned robotic platforms could in principle allow for autonomous operations as depicted in Figure 2, the current main challenges lie in the self-localization of the vehicles. No available localization approach provides satisfactory results for autonomous operations of μAUVs in confined tanks so far. Any kind of useful autonomous μAUV behavior requires the μAUVs to know their absolute positions. In the last few years, a very promising underwater localization framework based on the attenuation of electro-magnetic (EM) carrier waves in water was introduced in a series of papers [3,4,5,6,7]. In principle, the approach provides a satisfactory solution for the localization problem in confined tanks; however, it is not of immediate use for μAUVs. The method utilizes a signal analyzer that is too large and expensive to be included in a μAUV. This paper presents an embedded low-cost version of the EM localization principle for global self-localization of μAUVs.

### 1.1. Challenges in Underwater Localization in Confined Tanks

Outdoor robots usually rely on Global Navigation Satellite Systems (GNSS) for localization, while robots that are operated indoors, such as aerial vehicles, are localized with external camera systems. In contrast, submerged robots cannot use GNSS, because the signal is attenuated in water. Camera systems are also problematic, because image recognition underwater is difficult due to the demand for good visibility and ambient illumination. Furthermore, in order to obtain a global position based on vision, image-processing has to be performed off-board, and the pose has to be sent to the robot, as presented in [8]. Hence, communication bandwidth and computing capabilities limit the amount of robots that can be localized simultaneously with external camera systems. Off-board localization also introduces latencies and, thus, degrades closed-loop controller performances. One desirable capability of μAUV localization systems is therefore the ability to perform on-board self-localization. In [9], a vision-based SLAM algorithm is used to reconstruct 3D underwater environments. It is, however, still subject to the challenges of underwater camera systems. Furthermore, μAUV fleets with many members require a localization framework that is not affected by the number of fleet members. This can be achieved with a passive localization approach, i.e., an ambient reference signal that is observed without active transmission to beacons.

### 1.2. Related Work

Passive self-localization systems rely on a signal that is sent by beacons, which are installed prior to operations and whose positions are known to the mobile robots. The robot position is obtained in relation to the beacons by either determining the range to the beacons (spherical positioning) or by determining the difference between the ranges (hyperbolic positioning). Acoustic localization is one possible approach for confined underwater environments. However, in contrast to open waters such as oceans or lakes, multi-path propagation, reflections and reverberation of one to two seconds provide serious challenges for acoustic approaches. Moreover, μAUVs often do not possess the possibility for clock synchronization, which helps to accurately determine the time of flight of acoustic signals. An acoustic spherical positioning system with channel switching that does not require clock synchronization is suggested in [10]. The authors present simulations whereby the degrading effects are not included because they are in general difficult to simulate. In [11], an acoustic hyperbolic localization approach for confined environments is presented. Cyclically-transmitted signals render clock synchronization unnecessary. Experimental results show sub-decimeter localization accuracy, but vulnerability to reflections and reverberation. An acoustic modem for μAUVs that allows packet-based ranging and active localization is presented in [12,13]. The hardware is suitable for medium-sized (15 m to 100 m length scales) underwater environments.

Recently, a series of papers [3,4,5,6,7] by groups from the Pohang University of Science and Technology (POSTECH) and the Seoul National University of Science and Technology (SEOULTECH) introduced a novel approach for determining ranges underwater by using spherical localization based on the attenuation of EM carrier waves. Furthermore, the authors show the feasibility of the system for accurate localization in confined underwater environments. In [3] the authors derive the relationship between range and EM wave attenuation from the Maxwell equations and the Friis transmission formula. This relationship is used in [4] to demonstrate self-localization in the horizontal plane based on the attenuation of EM carrier waves. Further analysis of the system in freshwater and seawater is provided in [4]. Thorough analyses for the issues occurring, when the system is deployed in three-dimensional space, are provided in [6,7]. The results are used to show the feasibility of self-localization in three-dimensional space. The benefits of the EM approach are that multi-path propagation, reflections and reverberation are much weaker for EM waves than for acoustic waves in water because of the high attenuation rate in liquids. The localization accuracies that are reported in [3,4,5,6,7] are within millimeter range at a 1000-Hz update rate. A key step in the signal processing chain is the computation of a power density spectrum of an ultra-high frequency (UHF) signal. All results reported in [3,4,5,6,7] use a full-fledged spectrum analyzer for computing the power density spectrum. Such a spectrum analyzer is not deployable in μAUVs, because it is too large, too heavy and too costly. An embedded system that allows the installation on-board μAUVs will necessarily have a degraded performance in terms of localization accuracy and update rate. However, even accuracies of a centimeter magnitude at an update rate of 10 Hz would still fulfill most localization specifications for confined underwater environments, if the approach can be embedded within μAUVs. It would be a significant improvement over the state of the art, which would render μAUV operations in confined underwater environments possible.

### 1.3. Contributions and Outline

We extend the EM localization approach introduced in [3,4,5,6,7] to the μAUV domain by developing a small and low-cost embedded system architecture. The system is compact enough to be fit into the HippoCampus vehicle shown in Figure 1 and has a system price of less than USD 100. We demonstrate that the most critical step in the signal processing chain, the computation of a UHF power density spectrum, can be performed on a digital video broadcasting-terrestrial (DVB-T) receiver that is of similar size as a standard USB dongle. We provide thorough analysis of the system performance. This contribution intends to be a basis for implementing a low-cost underwater self-localization system based on off-the-shelf low-cost components.

The remainder of the paper is organized as follows. In Section 2, we recap the theoretical background of EM-based localization. Furthermore, we outline the signal processing algorithms for localization. Section 3 presents the hardware design, while Section 4 introduces the firmware. Experimental results for self-localization in a confined tank are presented in Section 5. The concluding remarks and an outlook to future work are presented in Section 6.

## 2. Theoretical Background for Spherical Localization Based on Attenuation of EM Waves

In this section, we present the theoretical background of the spherical localization systems. We first describe the spherical localization problem in general. Spherical localization requires range estimation between a receiver and beacons. The physical effects that enable underwater range estimation between the receiver and beacons are briefly summarized. The Cramér–Rao lower bounds are computed for the estimation problem to assess the accuracy of the localization system. An extended Kalman filter (EKF) and a particle filter (PF) are introduced, and either can fuse the ranges to obtain the receiver position.

### 2.1. Spherical Localization

Consider *N* beacons at known positions that emit EM waves of constant magnitude, with the *i*-th beacon located at position $r_{i}$. The receiver has the unknown position p.

The spherical self-localization problem can be decomposed into two parts: (1) range estimation from receiver node to transceiver beacons; (2) fusion of estimated ranges to obtain the position of the receiver. Spherical localization can be passive and is, hence, scalable within an increasing number of mobile receivers.

**Figure 3:**
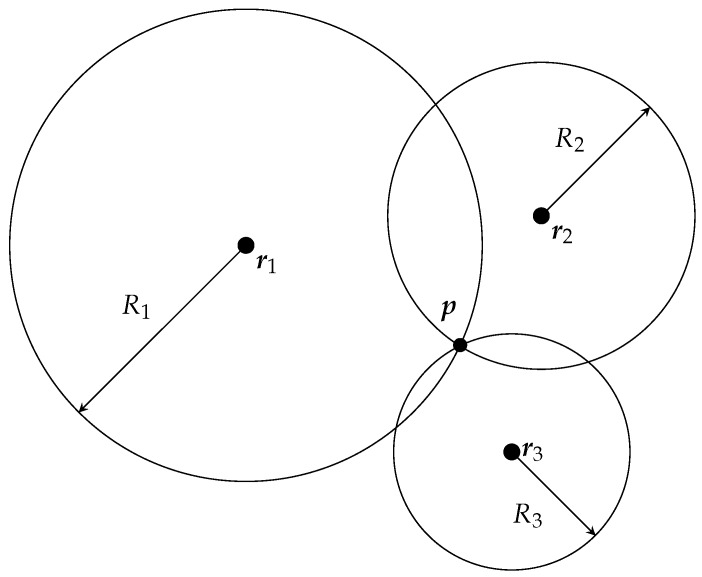
Spherical localization for determining the receiver position p as the intersection of circles in 2D or spheres in 3D of radius $R_{i}$. The known positions of the beacons are denoted by $r_{1}$, $r_{2}$ and $r_{3}$.

In spherical localization, the receiver node determines ranges from the receiver to beacons by measuring either the time of flight or the received strength of the signal that is emitted by the beacons. In the presented approach, the received signal strength (RSS) is measured. The range between the receiver and the *i*-th beacon can be expressed by:

(1) $$R_{i}=∥p-r_{i}∥,$$

which is the equation for a sphere. Hence, the ranges define spheres that are centered at the beacon locations. The intersection of all spheres is the receiver position. Consequently, the position of the receiver can be derived from geometrical relationships if the beacon locations are known as illustrated in Figure 3. Due to uncertainty in the measurement model and noise, the spheres do not intersect in general, and the intersection point needs to be estimated, e.g., with an EKF. In order for a receiver unit to self-localize in *n* dimensions, range estimations to at least n+1 beacons are required.

### 2.2. Underwater Range Sensor Model

We use a model called the underwater range sensor model (URSM) to estimate the range between receiver and beacons. The URSM describes the path loss attenuation as a function of distance. We use the model that was first introduced in [3]. The beacons transmit an EM signal with constant known power, and the RSS is measured at the receiver position. Based on the RSS, the URSM provides a range estimation between receiver and beacons. In air, the attenuation rate of EM waves is small over distance. This leads to large fluctuations in the RSS due to reverberation and interference. In contrast, EM waves in water attenuate rapidly with distance. Since disturbing effects such as reverberation or interference are less pronounced underwater, the principle of attenuating EM waves can be reliably used to estimate the distance from an EM transmitter to a receiver underwater. In order to map RSS values to range estimates, several techniques have been proposed in the literature. In air, the sensor model relies on the Friis transmission formula, which is commonly used to calculate ranges for a measured received signal strength. However, this model needs to be modified to consider medium parameters, such as temperature and conductivity. Park et al. [3,6] developed an EM wave attenuation model for underwater environments. The model is a modified version of the Friis transmission formula, which takes the attenuation constant α of the plane wave equation into account. The difference between the EM wave power $S_{R}$ on the receiver side and the EM wave power $S_{T}$ on the transmitter side is the RSS. The RSS as a function of the range $R_{i}$ between the receiver unit and the *i*-th beacon reads:

(2) $${\mathrm{RSS}}_{i}=S_{R,i}-S_{T,i}=-20\;\log_{10}\;R_{i}-20\;R_{i}\;\alpha_{i}\;\log_{10}e+\Gamma_{i}\;\left[\mathrm{dBm}\right]\;,$$

where $\Gamma_{i}$ is an offset factor representing antenna and environmental influences. The parameters $\alpha_{i}$ and $\Gamma_{i}$ can be calculated explicitly if variables such as polarization loss factor, transmitting and receiving antenna gains and the attenuation factor are known. In [3], these parameters are derived for an underwater test tank environment, and the resulting model is validated with experimental data. In our contribution, the model parameters are computed from spatially-distributed RSS measurements by fitting (2) with non-linear least-squares. The range $R_{i}$ as a function of $S_{R,i}$ can be obtained from (2):

(3) $$R_{i}=\frac{1}{\alpha \;\log_{10}e\;\ln10}\;W\left\{\alpha \;\log_{10}e\;\ln10\;\exp\left[\frac{-\ln10}{20}\;\left(S_{R,i}-S_{T,i}\right)-\frac{\Gamma \;\ln10}{20}\right]\right\}\;,$$

where W(x) is the Lambert-W function, which is the inverse function of:

(4) f(x)=xexp(x),

(5) $$f^{-1}\left(x\right)=f\left(y\right)=W\left(y\right)\;.$$

**Figure 4:**
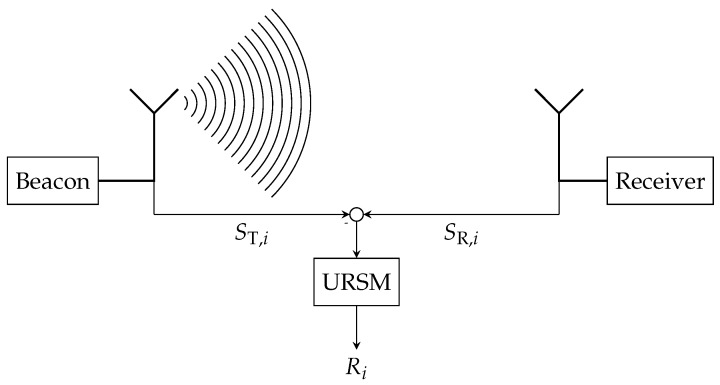
The underwater range sensor model (URSM) is applied to determine the range $R_{i}$ between the receiver unit and the *i*-th beacon. It maps the difference between received EM wave power $S_{R,i}$ and transmitted EM wave power $S_{T,i}$ to the range $R_{i}$.

The procedure of determining the range $R_{i}$ as a function of signal strength differences is depicted in Figure 4 for one dimension. The beacon emits an EM signal with the known power $S_{T,i}$, while the receiver measures the signal and determines the channel power $S_{R,i}$. The URSM allows computing the range between the beacon and receiver with (3).

### 2.3. Signal Identification Using Channel Allocation

Localization within a horizontal plane requires at least three beacons. An important aspect in RSS-based spherical localization is that the receiver has to assign the RSSs to the respective emitting beacons. In principle, a receiver can identify the signal source by time scheduling [14]. However, this requires clock synchronization, which is usually a challenging task for underwater applications [11].

**Figure 5:**
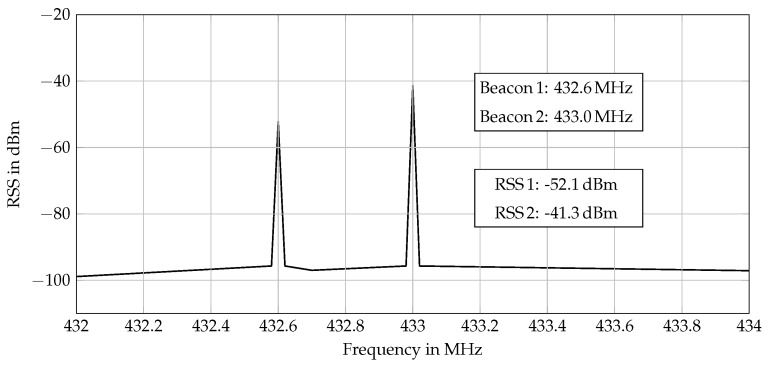
Identification of two beacons transmitting at 432.6 MHz and 433.0 MHz, respectively. The receiver unit identifies the beacons and corresponding RSS values by determining the frequencies and peak values.

An alternative approach for beacon identification is channel assignment, which is deployed in [4,5,6]. Hereby, each beacon sends an EM signal at a unique frequency. The resulting superposed signal is then measured by the receiver. By applying a fast Fourier transformation (FFT) on the receiver side, each RSS can be allocated to the respective beacon. This technique increases the update rate significantly, because beacons can transmit their signals simultaneously and do not need to wait for a scheduled time. Moreover, in each cycle, the FFT provides access to all RSS values. This is shown in Figure 5 for two beacons with different emitting frequencies. The prior knowledge about the beacons’ frequencies allows allocating the determined RSS values to the beacons and, thus, based on the corresponding URSM, to calculate the range between the each beacon and the receiver.

### 2.4. Cramér–Rao Lower Bound

The mobile receiver unit computes its ranges to the beacons by using the measurements of the RSS values on different channels. The ranges are then fused to estimate the receiver’s position, i.e., with a Bayesian filter, such as EKF or PF.

As a measure for the achievable accuracy of the localization system the Cramér–Rao lower bound (CRLB) can be computed to express a lower bound on the variance of the estimated receiver position. The CRLB is the inverse Fisher information matrix and can be computed as:

(6) $$\mathrm{CRLB}=F^{-1}.$$

Define a mean vector of received signal strengths:

(7) $$\mu ={\left[{\mathrm{RSS}}_{1}\;{\mathrm{RSS}}_{2}\cdots {\mathrm{RSS}}_{N}\right]}^{⊤}.$$

We assume the noisy signal strength measurements to be zero-mean Gaussian with covariance matrix $C=\mathrm{diag}(\sigma_{\mathrm{RSS},i}\left(R_{i}\right))$ and combined in the vector:

(8) τ∼N(μ,C).

The Fisher information matrix $F\in R^{n,m}$ has the elements:

(9) $$F_{m,n}=\frac{\partial \mu^{⊤}}{\partial p_{m}}C^{-1}\frac{\partial \mu }{\partial p_{n}}+\mathrm{tr}\left(C^{-1}\frac{\partial C}{\partial p_{n}}C^{-1}\frac{\partial C}{\partial p_{m}}\right).$$

The two-dimensional localization case yields $p_{m}=x$ and $p_{n}=y$.

**Figure 6:**
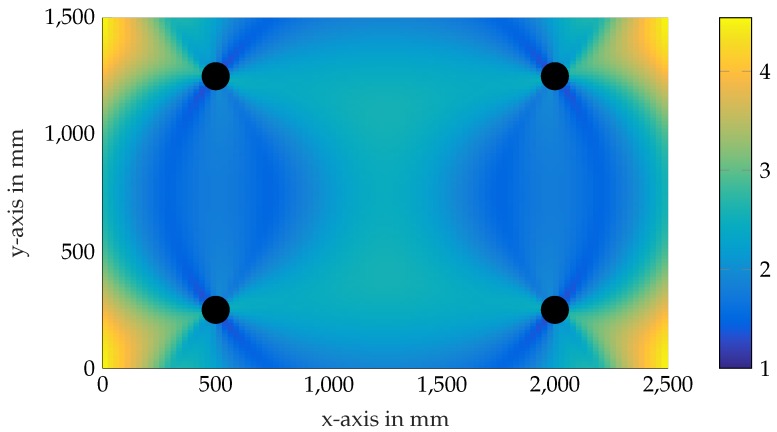
Normalized CRLB plot for a linearly-increasing measurement covariance function. Black markers depict the beacons.

The CRLBs for a localization scenario with four emitting beacons are illustrated qualitatively in Figure 6. Thereby, the covariance $\sigma_{\mathrm{RSS},i}\left(R_{i}\right)$ is modeled as a linearly-increasing function of the range to the *i*-th beacon. The CRLBs indicate that the lowest position estimation covariance lies within the convex hull spanned by the beacons.

### 2.5. Extended Kalman Filtering

In the following, an EKF algorithm is derived to estimate the position state vector p based on the RSS measurements. This approach allows for tightly-coupled sensor fusion, with, e.g., an IMU or a pressure sensor. We include the observation vector τ from (8) directly into the EKF algorithm. During the filter update step, each measured RSS value is processed independently. The loss of one or more RSS values still results in a position update along the remaining single spheres’ manifolds. This allows for optimal exploitation of the available information, and it improves the robustness of the system, because glitches in the EM measurement system can be treated by the EKF in a systematic way. The position state update is performed in every time step. In the following, superscripts (−) and (+) denote a value gained in the filter prediction or the filter update step, respectively.

We set the receiver’s state dynamics to be a random walk. Thus, the receiver’s state at time step *k* reads:

(10) p(k)=p(k−1)+w(k).

The process noise vector w is assumed to be zero-mean Gaussian white noise with covariance matrix Q. The non-linear measurement function μ(p) (7) defines a vector of RSS mean values as functions of the receiver position. The components ${\mathrm{RSS}}_{i}$ introduced by the URSM in (2) are updated by RSS measurements (8). The EKF requires the Jacobian $J_{\mu }\left(k\right)$ of μ(p), which consists of the derivatives $h_{i}^{⊤}\left(k\right)$ of (2):

$$h_{i}^{⊤}\left(k\right)=\nabla_{p}\;{\mathrm{RSS}}_{i}\left(p\left(k\right)\right)={\left[-\frac{20}{\ln10\cdot {∥p\left(k\right)-r_{i}∥}^{2}}\cdot {\left[p\left(k\right)-r_{i}\right]}^{⊤}-20\;\alpha_{i}\;\log_{10}e\frac{\left[p\left(k\right)-r_{i}\right]⊤}{∥p\left(k\right)-r_{i}∥}\right]}_{p={\hat{p}}^{\left(-\right)}\left(k\right)},$$

$$J_{\mu }\left(k\right)={\left[\begin{array}{c} h_{1}^{⊤}\left(k\right)\;\cdots \;h_{N}^{⊤}\left(k\right) \end{array}\right]}^{⊤}.$$

The predicted position state is:

$${\hat{p}}^{\left(-\right)}\left(k\right)={\hat{p}}^{\left(+\right)}\left(k-1\right)+w\left(k\right).$$

and its covariance reads:

$${\hat{P}}^{\left(-\right)}\left(k\right)={\hat{P}}^{\left(+\right)}\left(k-1\right)+Q.$$

The innovation and the innovation covariance are computed as:

$$\kappa \left(k\right)=\tau \left(k\right)-\mu ({\hat{p}}^{\left(-\right)}\left(k\right))$$

and:

$$S\left(k\right)=J_{\mu }\left(k\right){\hat{P}}^{\left(-\right)}\left(k\right)J_{\mu }^{⊤}\left(k\right)+C\left(k\right)\;$$

respectively.

The Kalman gain:

$$K\left(k\right)={\hat{P}}^{\left(-\right)}\left(k\right)\;J_{\mu }\left(k\right)\;S^{-1}\left(k\right)$$

allows one to compute the state update:

$${\hat{p}}^{\left(+\right)}\left(k\right)={\hat{p}}^{\left(-\right)}\left(k\right)+K\left(k\right)\kappa \left(k\right)$$

and the covariance update:

$${\hat{P}}^{\left(+\right)}\left(k\right)=\left(I-K\left(k\right)\;J\left(k\right)\right)\;{\hat{P}}^{\left(-\right)}\left(k\right).$$

### 2.6. Particle Filtering

As an alternative to the EKF, a PF can be used to estimate the position from the measured signal strengths (8). Particle filters (also referred to as sequential Monte Carlo filters) perform better than an EKF if the problem variance is large and the measurement model highly non-linear [15]. The sampling importance resampling (SIR) PF [16] is recapped in this subsection.

The distribution of the receiver position is approximated by a particle set consisting of *M* particles $\left\{p^{\left[m\right]}\left(k\right)\right\}$ indexed by the integer *m*. In addition, the particles are associated with weights $w^{\left[m\right]}\left(k\right)$, which indicate the importance of each particle. The weights are positive and are enforced to always sum up to unity, i.e., $\sum_{m=1}^{M}w^{\left[m\right]}=1$.

At time step *k*, a temporary particle set $\left\{{\tilde{p}}^{\left[m\right]}\left(k\right)\right\}$ is created from the particle set of the previous time step k−1 according to the random walk model (10):

(11) $${\tilde{p}}^{\left[m\right]}\left(k\right)=p^{\left[m\right]}\left(k-1\right)+w\left(k\right).$$

The unnormalized weight $w_{u}^{\left[m\right]}\left(k\right)$ is computed as the probability of the measurement τ(k) for particle ${\tilde{p}}^{\left[m\right]}\left(k\right)$:

(12) $$w_{u}^{\left[m\right]}\left(k\right)=N\left(\tau \left(k\right)|\mu \left({\tilde{p}}^{\left[m\right]}\left(k\right)\right),\;C\right).$$

In order to avoid particle degeneration, the temporary particle set is resampled. The weights $w_{u}$ are normalized to sum to unity, and all particles are drawn with replacement $p^{\left[m\right]}\left(k\right)∼\left\{{\tilde{p}}^{\left[i\right]}\left(k\right)\right\}$ with probability $\propto w^{\left[i\right]}\left(k\right)$, i=1,…,M. The position estimate $\hat{p}\left(k\right)$ can be obtained by averaging the resampled particles set:

(13) $$\hat{p}\left(k\right)=\frac{1}{M}\sum_{m=1}^{M}p^{\left[m\right]}\left(k\right).$$

## 3. Hardware Architecture

This section describes the main hardware components of the embedded underwater localization system. The beacons and the receiver unit are the main modules. In the following subsections, the underwater antenna design, the setup of the fixed beacons and the mobile receiver unit are introduced.

### 3.1. Antenna Design

**Figure 7:**
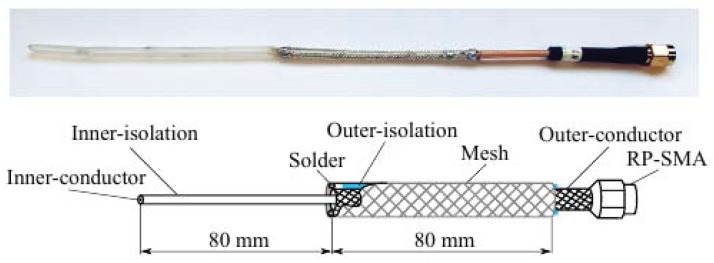
Photo (top) and schematic (bottom) of an underwater sleeve-dipole antenna for EM localization.

For localization within the horizontal plane, the signal must be emitted omnidirectionally in azimuth, in order to fulfill the system requirements for robustness and efficiency in an underwater environment. For this purpose, a half-wavelength dipole antenna was designed for underwater use, avoiding effects of air-water interfaces, as well as significantly reducing its length compared to terrestrial counterparts. A detailed build instruction is provided at https://youtu.be/muQLcZg0dWk. In consideration of good maintainability, simple sealing and future applications onboard μAUVs, the sleeve-dipole design is favored over a dipole T-shape. The underwater antenna is made of RG316 coax cable and matched to a 50Ω coaxial transmission line. The medium of propagation noticeably affects the signal wavelength and hence influences the antenna length. In the freshwater experimental environment, the best signal transmission is observed with an antenna length of 160 mm. Note, that the antenna length depends strongly on the conductivity of the surrounding water. The connection between antenna and feeder is realized through a dielectric tube in which an industrial PVC cable-sealing fixes the antenna. The antenna build-up is illustrated in Figure 7 with a photo and a schematic.

**Figure 8:**
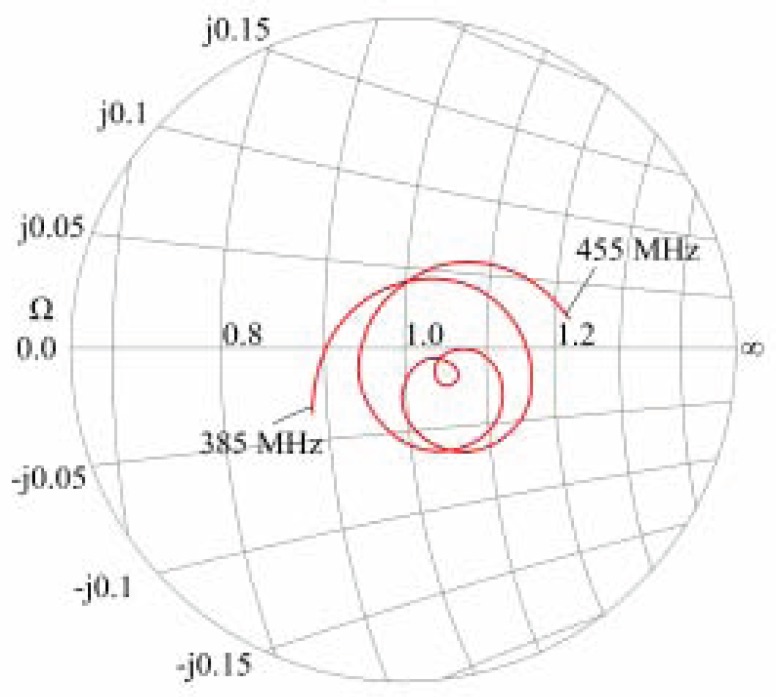
Smith chart of the underwater antenna.

Figure 8 shows the Smith chart of the deployed antennas in freshwater with a conductivity of σ=0.031S/m at 18 °C.

### 3.2. Fixed Beacons

**Figure 9:**
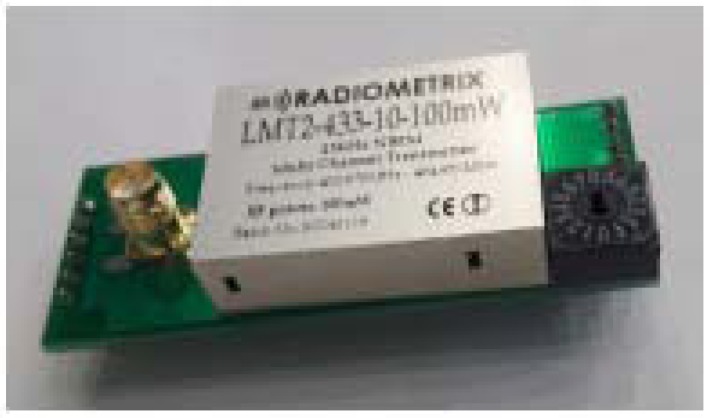
Circuit board with the Radiometrix^TM^ USX2 module for generating EM carrier waves at 433 MHz.

The beacons are anchored in the underwater environment and emit the EM signals. The signal-generating unit of each beacon is a custom-made circuit board with a Radiometrix^TM^ USX2 multi-channel half duplex UHF transceiver operating in the 433-MHz band. The circuit board is shown in Figure 9.

**Figure 10:**
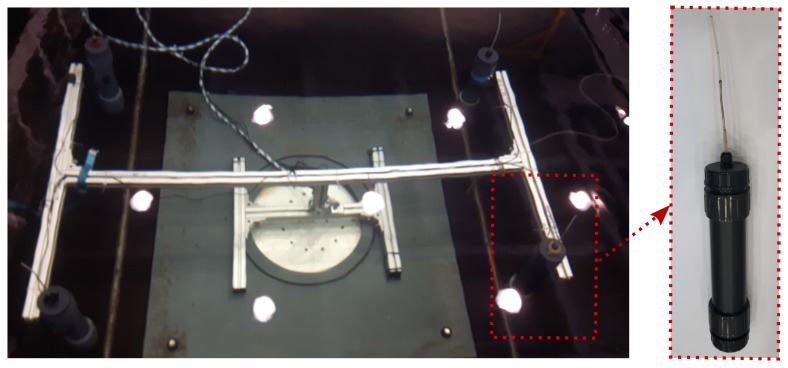
Submerged rack with four beacons. Each beacon contains a signal generating unit and continuously emits an EM signal at a unique frequency.

The beacons consist of the circuitry, which is housed in a sealed cylindrical polymer tube with the antenna being exposed to water. The UHF transceivers are powered by 12-V LiPo-batteries. In order to spatially fix the beacon configuration, they are arranged as an array on a frame. This allows placing the localization systems at almost any desired position in the work space. Figure 10 shows a submerged array of four beacons and a detailed photo of a single beacon.

### 3.3. Mobile Receiver Unit

The mobile receiver unit consists of an underwater antenna, a modified DVB-T USB dongle with the capability to compute a power spectrum density and a single board computer (SBC). The major design criteria for the module are size and cost, as it has to fit into space-constrained μAUVs such as the HippoCampus in Figure 1.

The mobile receiver unit carries out two main tasks:

1. it calculates real-time RSS values based on the URSM;
2. and it computes its position from the RSS values.

**Figure 11:**
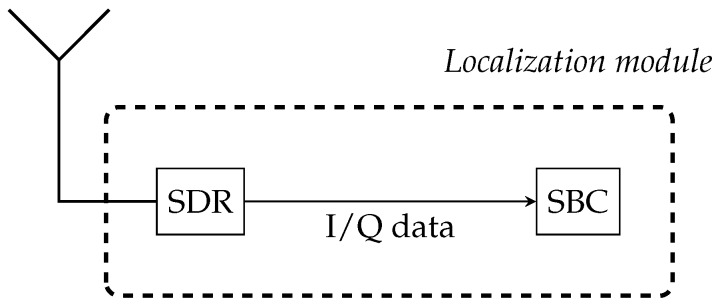
Onboard localization module: a software-defined radio provides I/Q samples to a single board computer.

The DVB-T USB dongle is used to digitize a segment of the EM spectrum as in-phase/quadrature (I/Q) samples. In order to drive the DVB-T USB dongle and to perform the RSS localization tasks, the SBC requests I/Q samples from the DVB-T USB dongle and computes the power density spectrum. The RSS values of the transmitter channels can be extracted from the spectrum and used for the range estimation. The functionality of the localization module is depicted in Figure 11.

#### 3.3.1. Modified DVB-T USB Dongle

**Figure 12:**
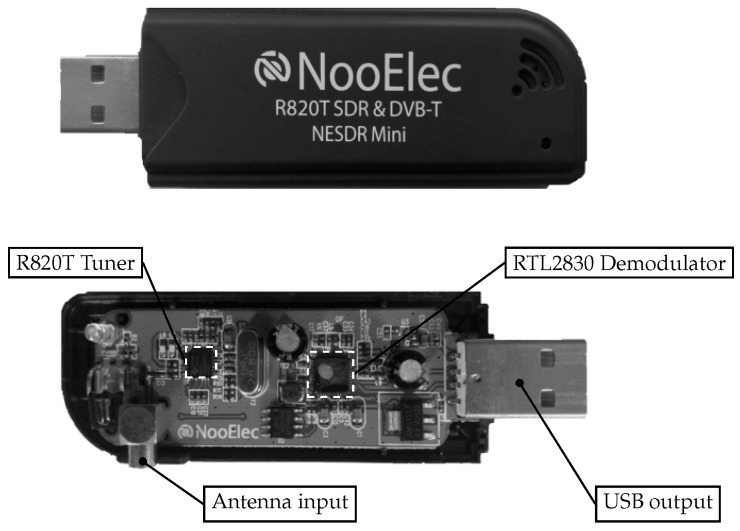
Covered and uncovered version of a NooElec^TM^ NESDR Mini DVB-T dongle used as an software-defined radio (SDR) with an R820 tuner, RTL2832 demodulator and USB interface.

Since the beacons transmit signals at different frequencies, a power density spectrum of the received signal has to be computed to determine the RSS values and to identify the corresponding beacons. In order to compute the spectrum, a low-cost DVB-T USB dongle capable of software-defined radio (SDR) is used. This is the main contribution to bring the system to the μAUV domain. In the original work [3,4,5,6,7], a full-fledged spectrum analyzer is deployed. Instead, the NooElec^TM^ NESDR Mini DVB-T device (approximately USD 25) is chosen in this work and is depicted in Figure 12. It processes signal sequences received through an antenna within a range of 24 to 1700 MHz. After demodulation and analog digital conversion (ADC), it transmits them via USB interface to the SBC. The core elements of the DVB-T dongle are the tuner and the demodulator. The integrated circuit tuner used in this work is an R820 chip. It receives analog EM signals, amplifies them and performs bandpass filtering. Afterwards, it down-converts the signal to a lower intermediate frequency (sub-sampling). This allows the subsequent eight-bit-ADC to sample at much lower sampling rates than the carrier frequency of the incoming analog RF signal. The demodulator, a RTL2832U chip, contains the ADC and encodes the signal to I/Q samples via coded orthogonal frequency-division multiplexing (COFDM). The I/Q samples are then processed by the SBC for spectrum analysis and RSS estimation. The maximum sample rate of the demodulator amounts to 3.2 MS/s. However, in order to avoid sample dropping and due to USB 2.0 data transfer restrictions, the sample rate is set to 2.4 MS/s.

#### 3.3.2. Single Board Computer

The localization algorithm runs onboard the mobile receiver unit. The single board computer needs to be compact enough to fit the geometrical constraints of μAUVs. Its main tasks consist of powering and interfacing the DVB-T dongle via the USB interface, running all required computations for localization and providing the position estimates to other modules. The SBC Raspberry Pi Zero is chosen as the computing unit. With a size of 66 mm × 30 mm × 5 mm and a weight of 9 g, it fulfills all physical requirements for the use in μAUVs. It has a single-core CPU, which runs at 1 GHz, and it has 512 MB of RAM. The Raspberry Pi runs Raspbian, a Debian-based operating system.

## 4. Firmware Design

The firmware controls the localization module and runs on the SBC. In order to obtain a position estimate of the mobile receiver unit from RSS values, the firmware executes all required tasks, e.g., power spectrum computation or Bayesian filtering. The open source firmware can be obtained at https://github.com/DanielDuecker/RF_Localization or https://github.com/EugenSol/RFLoc.

**Figure 13:**
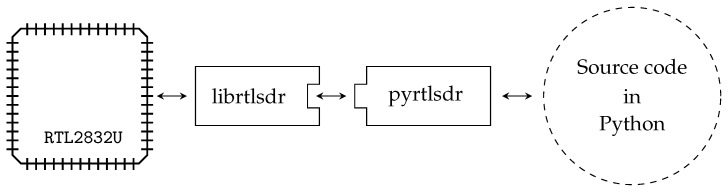
Communication between the digital video broadcasting-terrestrial (DVB-T) dongle and the single board computer (SBC). The RSL2832U chip-set is interfaced by the library librtlsdr. The wrapper pyrtlsdr provides convenient access to the library function. Both, librtlsdr and pyrtlsdr run on the SBC.

The Python programming language is chosen for the firmware implementation, because it is a widely-available cross-platform open-source language. The DVB-T dongle is interfaced via the librtlsdr library (https://github.com/librtlsdr/librtlsdr), which provides drivers for the RTL2832U chip-set. The wrapper pyrtlsdr (https://github.com/roger-/pyrtlsdr) is included, as well, as it conveniently wraps functions of the librtlsdr library to make them accessible within the Python software architecture. These functions include setting the center frequency, i.e., 433 MHz, on the DVB-T dongle and the access to the complex eight-bit I/Q samples, which are provided as an array for further signal processing. The communication chain is shown in Figure 13.

The localization process and, thus, the firmware are split into two main workflows: first, the calibration, which performs the parameter identification of the URSM, and second, the localization, which contains the localization algorithm via Bayesian filtering. Both steps are part of the super class RfEar-provided functionalities, which are commonly used for its subclasses CalEar and LocEar.

### 4.1. Calibration

The calibration process identifies the URSM parameters α and Γ for each beacon. It is organized within subclass CalEar and consists of three steps:

1. Measurement of the power spectrum density of the EM-field at a series of different positions, i.e., a grid.
2. Determination of RSS values for each beacon frequency by applying an FFT on the measured power spectrum density at each measurement position.
3. Fitting of the URSM for each beacon according to the collected data by using a non-linear least-squares algorithm.

### 4.2. Localization

The subclass LocEar is used to execute the localization of the receiver unit based on measured RSS values. Thus, the URSM parameters must be determined in advance through calibration. In order to localize the position of the receiver unit, different estimation algorithms are implemented, i.e., EKF and PF, which fuse the measured RSS values to an estimated position of the receiver unit. The estimated position and its uncertainty can then be transmitted to other algorithms, i.e., to allow closed-loop trajectory following.

## 5. Results

This section presents experimental results to validate the feasibility and performance of the embedded RF localization system in water. We analyze performance tradeoffs arising due to data processing with the NooElec^TM^ NESDR Mini DVB-T dongle instead of a full-fledged signal analyzer. We show localization results for static position hold and dynamic position estimation along trajectories.

### 5.1. Experimental Setup

**Figure 14:**
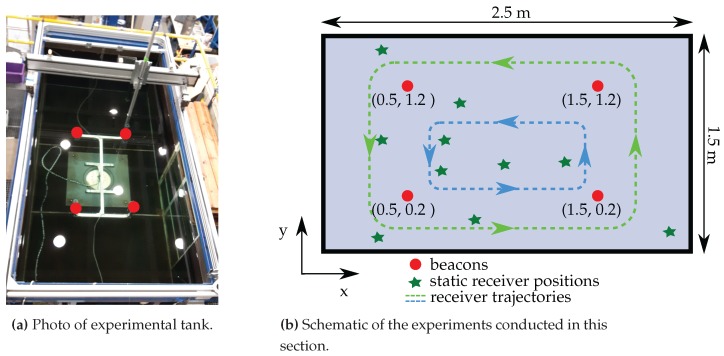
Experimental setup to validate the embedded EM localization system.

All experiments were carried out in a water tank available at the Institute of Mechanics and Ocean Engineering, Hamburg University of Technology. The experimental setup and the performed experiments are shown in Figure 14. In Figure 14b, the dashed lines depict the receiver unit trajectories and the stars the static positions. The conductivity of the water was measured and amounts to $0.031\;\frac{S}{m}$. Four EM beacons, as introduced in Section 3.2, are deployed in the experimental tank as shown in Figure 14.

The four beacons transmit EM signals at 433.90 MHz, 434.05 MHz, 434.20 MHz and 434.35 MHz. The receiver unit is mounted to a horizontal movable gantry unit with a workspace of 3 m by 1.6 m. This allows moving the receiver unit along pre-programmed trajectories and taking signal strength measurements with arbitrary high geometric resolution.

During static measurements, the localization system is able to reach sampling frequencies of 20 Hz. For dynamic measurements, the sampling frequency is approximately 4.5 Hz. This is mainly due to the long response time of the gantry position encoder. It is worth mentioning that the sampling frequency is independent of the number of emitting beacons, since the algorithm executes an FFT for the relevant part of the power density spectrum, which includes all transceiving frequencies of the beacons.

### 5.2. Data Processing

**Figure 15:**
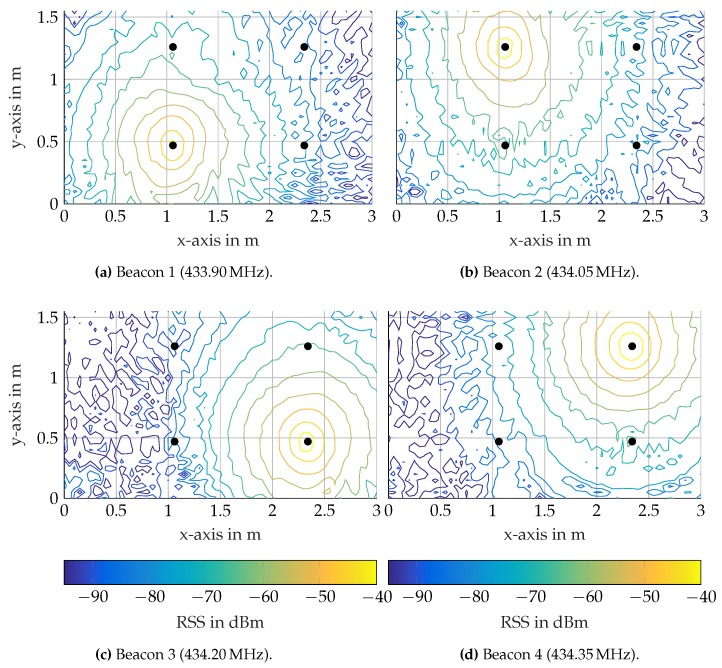
Contour plots of RSS values measured over 5 s on each grid point at the beacons’ frequencies. Black markers depict the beacons.

The presented setup allows pre-programing the motion of the receiver unit and capturing RSS values on a fine grid that is spread in the test tank. This is useful to study the EM field in the workspace. It allows analyzing tank specific field characteristics like reflections and noise. The measured signal strengths at the four different frequencies (433.90 MHz, 434.05 MHz, 434.20 MHz, 434.35 MHz) are illustrated as contour plots in Figure 15. The measurements were taken on a grid with a 50 mm by 50 mm resolution, where each of the 1952 grid points initiated a 5-s motion-free measurement sequence. The contour plots show the mean values of these measurement sequences. In each of the subfigures, the RSS is largest at the position of the respective beacon and decreases with distance, as expected. For the case of an ideal omnidirectional antenna, the potential lines of equal RSS values form circles. All beacons demonstrate this characteristic. However, with increasing distance from the beacons and in the vicinity of the tank walls, the circular contours ravel out. This is due to the noise floor level, which the RSS measurements reach, which begins for our system at approximately −85 dBm. The disturbances close to the tank walls are due to reflections from the tank walls, which are made of steel beams, wood and glass. The contour lines in Figure 15b are elliptically shaped around Beacon 2. This is likely due to an antenna mounting error, which results in a slight tilt of the antenna. Thus, the emission plane of the antenna is not aligned with the horizontal measurement plane of the receiver node.

An important aspect in the context of RSS data processing is the dynamic range of the receiver unit. The dynamic range is a measure for how well the weak signals can be detected in the presence of stronger signals on neighboring frequency channels. Hence, high dynamic range is desirable as it allows one to detect signals from far away beacons in the vicinity of other beacons. The NooElec^TM^ NESDR Mini DVB-T dongle has a dynamic range of approximately 60 dBm.

**Figure 16:**
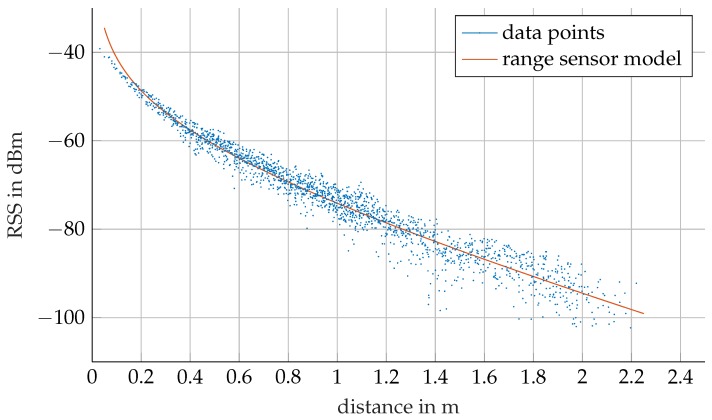
Underwater range sensor model and RSS measurements.

The RSS data can be used to validate the URSM (2) introduced by [3] first and summarized in Section 2.2. The measured data presented in Figure 16 are used to fit $\alpha_{i}$ and $\Gamma_{i}$ of (2). Figure 16 illustrates the measured RSS values as a function of distance for a single frequency and the fitted URSM. The data correspond to the analytical model, whereby the scattering increases with distance. Moreover, the measurements deviate from the URSM in direct vicinity of the emitting beacon. This effect is due to a vertical offset between the horizontal transmission plane of the beacon and the receiver node. For distances of more than 1.8 m the RSS measurements deviate significantly from the URSM, as the RSS reaches the noise floor level of the receiver unit. There are two options to resolve the effect with modifications of the beacons: first, increasing the power of the emitter and, second, using antennas with higher directivity. Both will increase average RSS in the test tank and, thus, push the noise floor to further regions. Another option is to add more beacons to the test tank, i.e., a total of six beacons arranged in a three by two pattern. In this case, the localization system does not need to rely on the RSS from far away beacons, as there are closer beacons whose EM signals allow more accurate RSS measurements. However, this approach would require a distance-dependent weighting of the RSS measurements in the filter algorithm to reduce the influence of inaccurate RSS measurements on the localization.

### 5.3. Static Position Estimation

**Figure 17:**
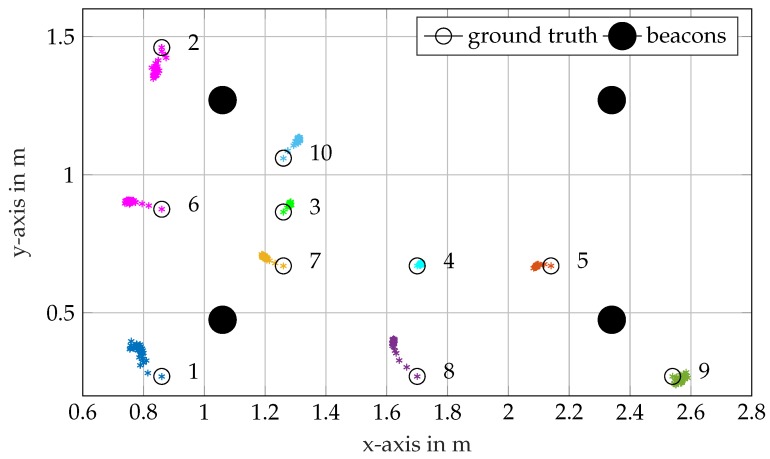
Static receiver self-localization at ten different positions. A PF with 1000 particles merges the measurements.

For the static position estimation experiment, ten positions with known ground truths are chosen. The receiver unit is placed at those positions, and the receiver positions are estimated with the localization system. Figure 17 shows the results for those positions, whereby the estimation is performed with a PF and M = 1000 particles. While Park et al. [4] report root-mean-square errors between 1 mm and 2 mm for their original method, the errors in Figure 17 are an order of magnitude higher. The data processing capabilities of the hardware are inferior to the signal analyzer in [4]. However, it is compact and inexpensive. Another reason for the degraded performance is the strong reflections in the tank. The points in proximity to the tank walls, e.g., 1, 2, 3 and 9, show systematic biases due to reflections, whereas results for points further away are more accurate, e.g., Points 3 and 4. Despite their biases, all measurements show a small variance. This emphasizes our assumption that the deviations mainly result from the reflections and are, thus, caused by the characteristics of our test basin, which is significantly smaller than the test tank used in [4].

### 5.4. Dynamic Position Estimation

**Figure 18:**
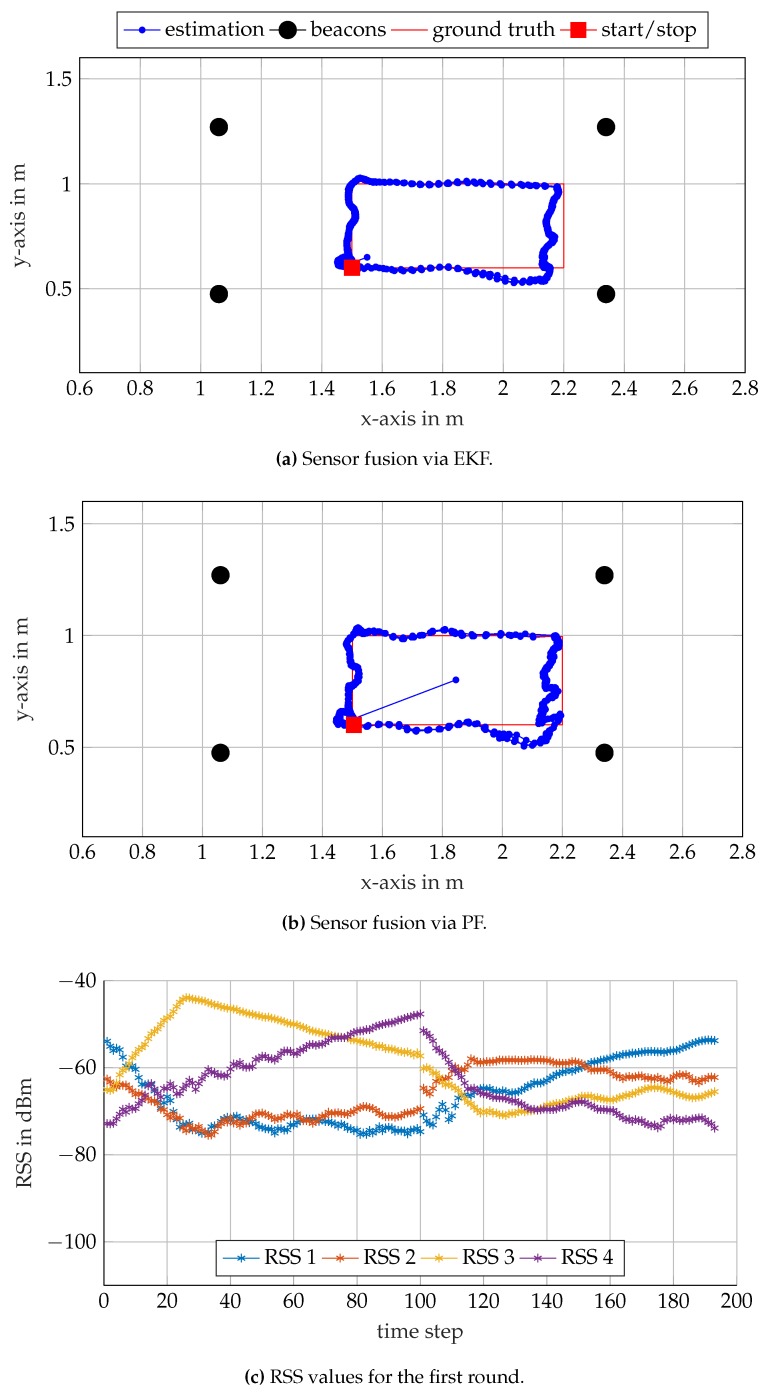
Self-localization results along a rectangular trajectory within the convex hull of the beacons. The receiver traverses the rectangle three times.

**Figure 19:**
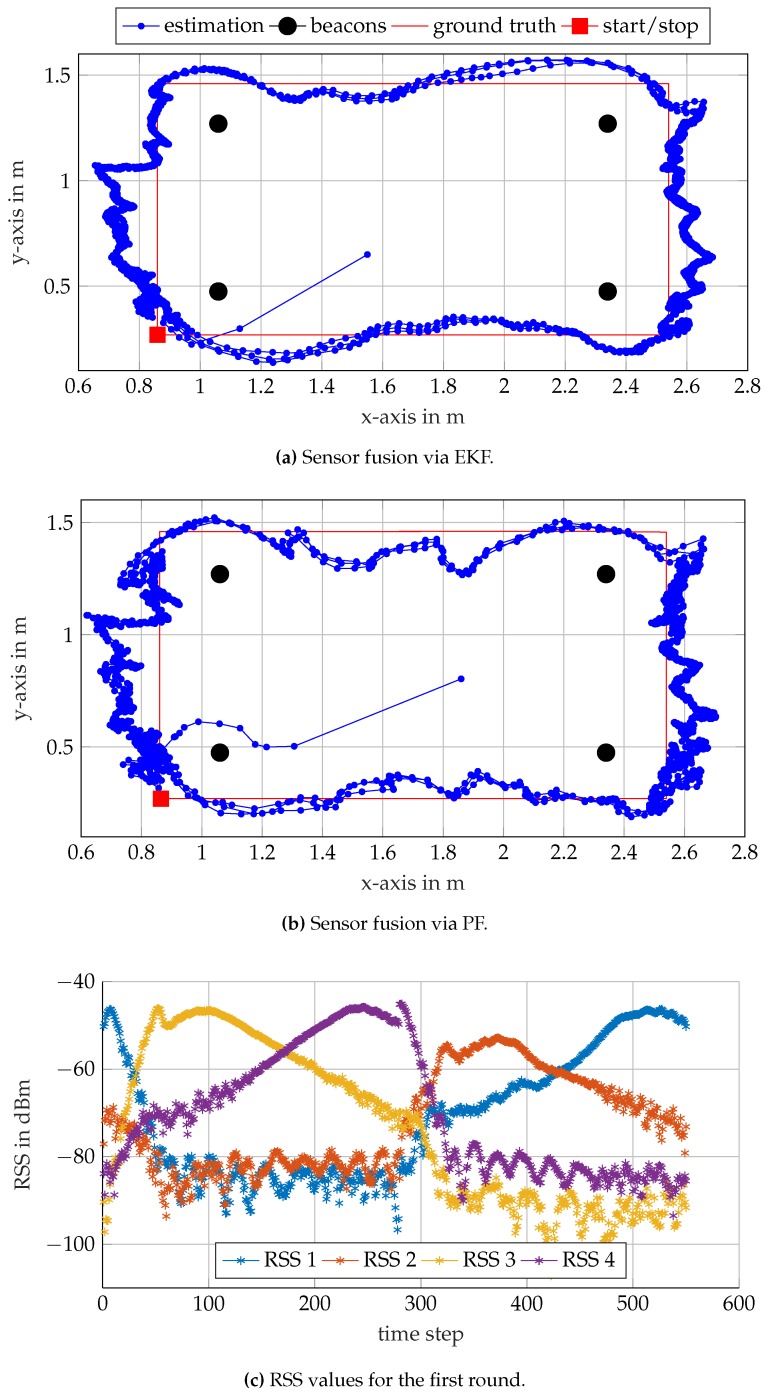
Self-localization results along a rectangular trajectory outside the convex hull of the beacons. The receiver traverses the rectangle three times.

In the final experiment, the receiver unit is guided along two different rectangular trajectories similar to the one in [4], albeit smaller. The first trajectory lies completely within the convex hull spanned by the beacons. The second trajectory lies outside of the convex hull. The results for the two trajectories are shown in Figure 18 and Figure 19. For each of the trajectories, an EKF and a PF are used to fuse the RSS values for position estimation. The ground truths and the estimated positions of the receiver are shown in Figure 18a,b for the inner and in Figure 19a,b for the outer rectangle. The RSS measurements at the four distinct frequencies are illustrated in Figure 18c and Figure 19c. As in the previous subsection, the results are less accurate than the ones reported in [4]. However, for most μAUV applications in confined test tanks, the results are sufficient, especially given the small size and cost of the system.

The EKF and PF approaches show similar results for both trajectories. In general, PFs tend to outperform EKFs if nonlinearities are strong and noise variance is large. For these cases, the Gaussian assumption on noise of the EKF framework is not accurate enough anymore. The PF allows including uniform priors, which can be an advantage in certain applications. It is worth mentioning that coupling with other sensors such as IMUs can be more straight forward for EKF’s framework than for PF’s.

The estimated positions are compared against the ground truth, and the RSS measurements are assigned to corresponding gantry positions. The RSS values in Figure 18c and Figure 19c exhibit discontinuities at Time Steps 100 and 300, respectively. At this point, the gantry did not provide ground truth data, and the RSS measurements were rejected for several consecutive time steps. Nevertheless, the system is able to recover the position as soon as the RSS measurements become available again, which demonstrates the robustness of the system.

## 6. Summary and Outlook

In this paper, we presented a compact, low-cost localization architecture based on the EM wave attenuation principle. The system provides satisfying performance with an accuracy in the centimeter range at a fraction of the cost and size of existing systems. Our main contribution is the extension of the system introduced in [3,4,5,6,7] to the μAUV domain.

The main system components are anchored beacons emitting EM signals in a frequency band centered around 433 MHz and a passive mobile receiving unit, which measures the RSS values to estimate its position relative to the beacons. The hardware part of the signal processing chain was realized with an off-the-shelf USB DVB-T dongle capable of sampling parts of the EM spectrum and an SBC, in this contribution a Raspberry Pi Zero. An FFT was implemented on the SBC to determine the RSS from each beacon within the received power density spectrum. Calibration allows us to fit the two parameters of the URSM to the RSS values and the distances between the mobile receiver unit and the corresponding beacons. The measured RSS values were fused to estimate the position of the receiver unit based on the RSM by deploying Bayesian filtering algorithms, such as EKF. Since the receiver unit is passive, only one-way signal transmission is required, and the system is not affected by the number of fleet members.

We demonstrated the performance of our architecture in experiments for static and dynamic applications and analyzed the EM field characteristics of the test tank. The experiments have shown an accurate and reliable localization of the receiver unit. However, the accuracy depends highly on the receiver’s position within the test tank. The results show that within the convex hull of the beacons, the localization is very accurate, whereas the accuracy decreases with larger distances from beacons and also in the vicinity of the tank walls. As the static position measurements have shown very small variances, we assume that the localization accuracy would improve significantly in a larger test environment, as inhomogeneities of the EM field that are mostly due to reflection from the tank walls would be reduced.

Further investigations should be conducted on the EM field characteristics. This includes the influence and improvement of antenna design. In order to analyze the system performance independently from the tank characteristics, we plan to deploy our system in a larger water tank with characteristic lengths of approximately 5 to 10 m to achieve a better homogeneity of the EM field. Moreover, we plan to extend the system capabilities to enable 3D localization, which is challenging due to the directivity of the antennas. Therefore, a possible option is to use the approach of [6] and extend it to μAUVs. It adds more beacons to the system and stacks them in multiple horizontal planes. The received measurement signals are to be fused together with the actual depth measurement of the μAUV to obtain the absolute position in 3D space. The goal is to embed the system in μAUVs and perform closed-loop position control.

## Abbreviations

The following abbreviations are used in this manuscript:

μAUVs	Micro autonomous underwater vehicles
ADC	Analog digital conversion
COFDM	Coded orthogonal frequency-division multiplexing
CRLB	Cramér–Rao lower bound
DVB-T	Digital video broadcasting-terrestrial
EKF	Extended Kalman filter
EM	Electro-magnetic
FFT	Fast Fourier transformation
GNSS	Global Navigation Satellite Systems
I/Q	In-phase and quadrature
PF	Particle filter
RF	Radio frequency
RSM	Range sensor model
RSS	Received signal strength
SBC	Single board computer
SDR	Software defined radio
UHF	Ultra-high frequency
USB	Universal serial bus
